# Treatment Differences for Splenic Flexure Cancers in Saudi Arabia: A Cross-Sectional Study

**DOI:** 10.7759/cureus.63821

**Published:** 2024-07-04

**Authors:** Abdulrahman Alotaibi, Abeer Zakariyah, Abdullah Malaka, Mohammad Alamri, Wajd Aljohani, Almaha Alshehri, Esraa Alghamdi, Nouf Almalki

**Affiliations:** 1 Colorectal Surgery, University of Jeddah, Jeddah, SAU; 2 Colorectal Surgery, Dr. Soliman Fakeeh Hospital, Jeddah, SAU; 3 Medical Genetics, University of Jeddah, Jeddah, SAU; 4 Surgery, University of Jeddah, Jeddah, SAU; 5 Internal Medicine, King Abdullah Medical Complex, Jeddah, SAU

**Keywords:** treatment, saudi arabia, surgeons, splenic flexure, cross-sectional study, colon cancer, colonic neoplasms

## Abstract

Backgrounds

Colorectal surgeons worldwide have differing opinions on the best way to handle rare cases of splenic flexure colon cancers (SFCs). Although the majority of reviews indicate no significant variation in oncological outcomes among the three different procedure types used to treat SFCs, surgeons still exhibit diversity in their practices. This study determined the treatment preferences of colorectal surgeons in Saudi Arabia.

Methods

A descriptive cross-sectional study evaluated the management of colorectal surgeons in handling SFC cases. We utilized a validated questionnaire developed by Manceau et al., consisting of 14 questions. Emails and phone numbers of members of the Saudi Society of Colorectal Surgery (SSCRS) were gathered. Google Forms surveys were administered from October 1-30, 2023.

Results

A response rate of 66% (58/88) was obtained among questioned colorectal surgeons. Their responses revealed that there was no consensus regarding the preferred procedure to treat SFCs. The most common treatment reported was segmental colectomy (SC) 21/58 (36.2%), followed by subtotal colectomy (STC) (19/58, 32.8%) and left hemicolectomy (LHC) (18/58, 31%). There was a strong consensus of 96% (56/58) of the respondents in favor of using stapler anastomosis rather than hand sewing. The frequency of performing SC, STC, and LHC in France was 70%, 13%, and 17%, respectively, compared to 36.2%, 32.8%, and 31% in Saudi Arabia, with a p-value of 0.001. The surgeons' preferred approaches to managing SFCs utilizing laparoscopic, open, or hand-aided in France versus Saudi Arabia were 63%, 31%, and 11%, respectively, compared to 84.5%, 8.6%, and 6.9%, with a p-value of 0.001.

Conclusion

A significant disparity exists regarding the treatment of SFCs between colorectal surgeons in France and Saudi Arabia. Furthermore, there is a lack of consensus among colorectal surgeons in Saudi Arabia regarding the surgical management of SFCs. Hence, it is imperative for the SCRSS to assemble a panel of experts to reach a consensus for the most appropriate and effective treatment of SFCs.

## Introduction

More than 935,000 people die annually from colorectal cancer (CRC), making it the third most common form of cancer overall and the second deadliest [1]. CRCs are detected in the rectum in approximately one-third of patients, with detection in the colon in the remaining two-thirds of patients.

Splenic flexure cancers (SFCs) are tumors located between the distal third of the transverse colon and the proximal descending colon. A small percentage of all CRCs are due to SFCs, accounting for less than 3% of cases [2]. A high obstruction risk is associated with SFCs, as the tumors present late, and the chances of curative resection are low [3-5]. Surgical resection is the primary treatment for all types of CRC. The extent of resection is determined by the location and lymphovascular drainage of the tumors [6]. As SFCs are located between the midgut and hindgut, their lymphatic drainage and vascular supply vary. It is further complicated by the participation of both the inferior and superior mesenteric vessels in the drainage of SFCs, which makes it difficult to standardize its surgical management [3].

Various surgical approaches have been used to treat SFCs, including subtotal colectomy, extended right hemicolectomy, left hemicolectomy, and splenic flexural colectomy. Several studies have examined the oncological and functional outcomes of different surgical approaches and have determined the preferred approach by surgeons [7-10]. However, there is a lack of relevant data from Saudi Arabia. Therefore, this study examined how CRC surgeons manage SFCs in Saudi Arabia.

## Materials and methods

This descriptive cross-sectional study assessed whether there are variations in the management of SFCs among colorectal surgeons in Saudi Arabia. The questionnaire on the management of splenic SFCs was derived from a validated one used by Manceau et al. [10] The questionnaire consists of 14 inquiries specifically targeting the professional activities of colorectal surgeons (see Appendix). The Bioethics Committee of Scientific and Medical Research of the University of Jeddah provided approval for this study (No. UJ-REC-130).

The email addresses and contact numbers of colorectal surgeons who are members of the Saudi Society of Colon and Rectal Surgery (SSCRS) were gathered. They were then requested to participate in an online survey using Google Forms (Google LLC, Mountain View, California, United States) from October 1 to October 30, 2023. Fourteen variables were analyzed with descriptive statistics to calculate frequencies and percentages to summarize the available data. Data are presented as tables and graphs.

Manceau et al. [10] undertook research to document the surgical techniques utilized by colorectal surgeons in France for SFCs. The study included the involvement of 190 surgeons, which accounted for 45% of the total 420 surgeons. Inferential statistics were used to compare the results of this study with those of Manceau et al. [10] Chi-square and Fisher’s exact tests were used to compare the results between the two studies, as all variables involved were categorical. Statistical significance was set at p < 0.05, and the confidence interval (CI) was set at 95%. Data were analyzed using IBM SPSS Statistics for Windows, version 25.0 (released 2017, IBM Corp., Armonk, NY). Moderate or strong consensus was reached when more than 50% or 70% of the participants agreed, respectively.

## Results

Surgery types performed among colorectal surgeons in Saudi Arabia for SFC

**Figure 1:**
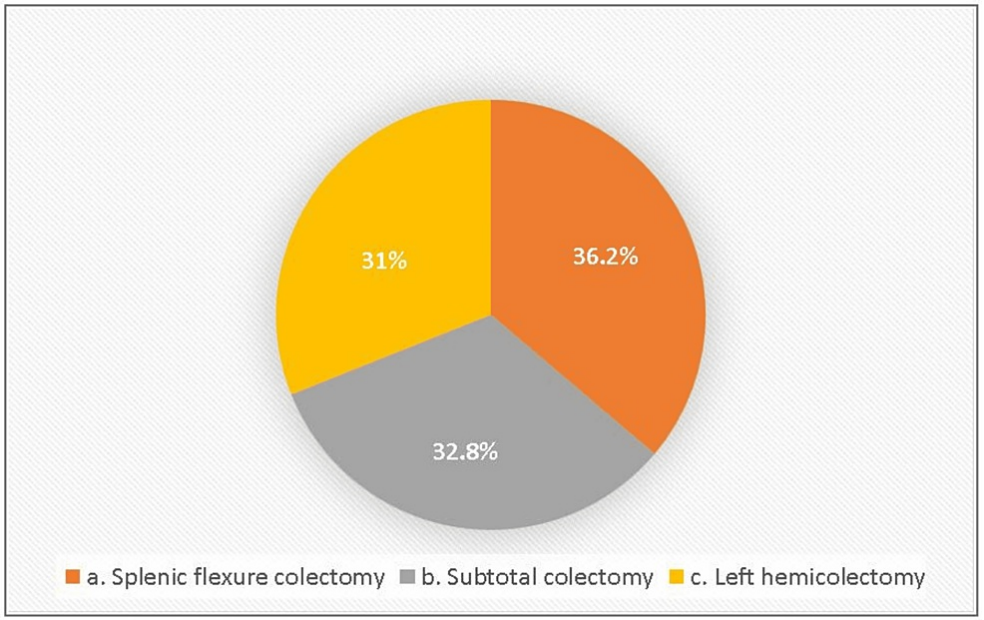
Types of surgery performed for splenic flexure colon cancers (SFCs) among colorectal surgeons in Saudi Arabia

The current study found that splenic flexure colectomy was the most common type of colectomy performed by colorectal surgeons in Saudi Arabia (36.2%, n = 21/58), followed by a subtotal colectomy (32.8%, n = 19/58) and left hemicolectomy (31%, n = 18/58). There was no agreement among colorectal surgeons in Saudi Arabia regarding the procedure to treat patients with SFCs (Figure 1).

Ligation of blood vessels and lymphadenectomy

**Figure 2:**
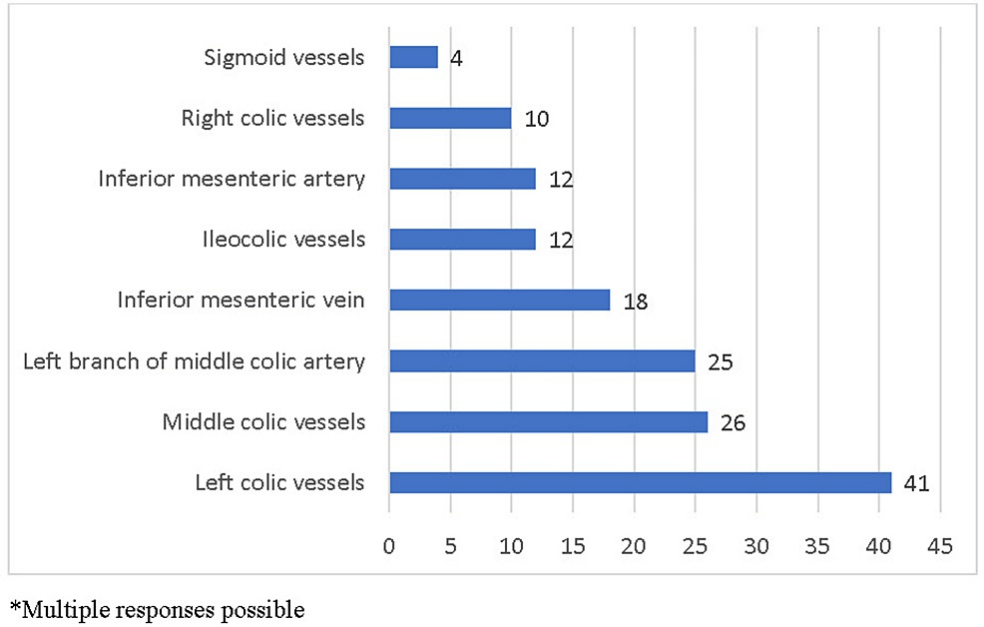
Ligation of different blood vessels reported by colorectal surgeons during the splenic flexure colon cancer (SFC) procedure.

In our study, most surgeons (70.68%, n = 41) reported ligating the left colic vessels at their origin during the procedure. Approximately 44.82% (n = 26) of the surgeons reported ligating the middle colic vessels, and 43.10% (n = 25) reported ligating the left branch of the middle colic artery during the SFC procedure. Other blood vessels ligated by surgeons during the SFC procedure are shown in Figure 2. Most surgeons did not routinely follow a specific method to assist with the lymphadenectomy (93.1%, n = 54). Only 6.9% (n = 4) of the surgeons reported that they had followed CT with vascular reconstruction for lymphadenectomy during the procedure for SFCs.

Anastomosis fashion

At the end of the procedure, most surgeons (43.1%, n = 25) reported performing anastomosis of the transverse colon with the descending colon. Approximately 25.9% (n = 15) of the surgeons reported an anastomosis between the transverse colon and upper rectum. Other anastomosis types between different digestive segments are shown in Table 1. The stapled side-to-side anastomosis was most frequently performed (48.3%, n = 28), followed by the stapled end-to-end anastomosis (31%, n = 18). Stapled side-to-end anastomosis and hand-sewn end-to-end anastomosis were reported in 17.2% (n = 10) and 3.4% (n = 2) of the patients, respectively. There was a strong consensus among the respondents (96%, 56/58) in favor of using a stapler anastomosis rather than hand sewing. Moderate agreement was observed for a preference for the stapled side-to-side method (48.3%, 28/58) among the other anastomosis configurations. The different types of anastomoses reported by the surgeons are presented in Table 2.

**Table 1:** Surgeons' choice for anastomosis of digestive segments during splenic flexure colon cancer (SFC) surgery (n = 58).

Anastomosis	Number	Percentage
Transverse colon and descending colon	25	43.1
Transverse colon and upper rectum	15	25.9
Terminal ileum and descending colon	8	13.8
Terminal ileum and sigmoid colon	6	10.3
Terminal ileum and upper rectum	4	6.9

**Table 2:** Types of anastomoses reported during the SFC procedure (n = 58)

Anastomosis procedure	Number	Percentage
Stapled side-to-side	28	48.3
Stapled end-to-end	18	31.0
Stapled side-to-end	10	17.2
Hand sewn end-to-end	2	3.4

Surgical approach and omentectomy

Among the surgeons, laparoscopy was the preferred surgical approach for performing procedures on patients with SFCs (84.5%, n = 49). This was followed by open surgery (8.6%, n = 5) and hand-assisted laparoscopy (6.9%, n = 4), as shown in Table 3. A moderate consensus of the surgeons (58.6%, 34/58) preferred performing a left partial omentectomy. Total omentectomy, which preserves the vascular perigastric arcade was performed in 22.4% (n = 13) of the patients, and complete preservation of the greater omentum was performed in 17.2% (n = 10). In addition to the above procedures, intraoperative colonic irrigation was reported by 3.4% (n = 2) of the surgeons and diverting stoma by 1.7% (n = 1) of surgeons. Approximately 94.8% (n = 55) of the surgeons did not perform any additional procedures during surgery. Internal herniation following laparoscopic left hemicolectomy was reported by 10.3% (n = 6) of the surgeons, while 89.7% (n = 52) reported no internal herniation following laparoscopic left hemicolectomy.

**Table 3:** Preferred surgical approach by colorectal surgeons in Saudi Arabia for doing splenic flexure colon cancer (SFC) procedure (n = 58)

Surgical approach	Number	Percentage
Laparoscopy	49	84.5
Open surgery	5	8.6
Hand-assisted laparoscopy	4	6.9

Tumor prognosis

Among the 58 surgeons who participated in this study, 31 (53.4 %) felt that the patients with SFCs had a worse prognosis than those with other colonic tumors. By contrast, 46.6% (n = 27) believed that patients with SFCs did not have a worse prognosis. Common reasons for a worse prognosis of patients with SFCs, as reported by surgeons, included diagnosis at a more advanced stage (36.2%, n = 21) and insufficient lymph node dissection (29.3%, n = 17). Other less common reasons included dual blood supply, histological type, and staging owing to lymph node skip metastasis.

Surgical procedures to treat obstructing SFCs

Approximately 34.5% (n = 20) of the surgeons performed segmental left colectomy for left-sided colonic cancer obstruction without evidence of proximal colon dilation or ischemia. For the resection of the obstruction of the left colon cancer, 69% (n = 40) of the surgeons preferred to cover the stoma. Anastomosis and on-table lavage were preferred by 25.9% (n = 15) and 5.2% (n = 3) of surgeons, respectively. Other surgical procedures for obstructing left-sided colon cancer are shown in Table 4.

**Table 4:** Surgical procedures performed by colorectal surgeons in Saudi Arabia for obstructing left-side colonic cancer (n = 58)

Surgical procedure	Number	Percentage
Segmental left colectomy	20	34.5
Resection with stoma	17	29.3
Extended right colectomy	15	25.9
Colostomy	6	10.3

Comparison of the study results with the results of the study done by Manceau et al. [10]

Manceau et al. [10] conducted a study with the aim of reporting the surgical practices done by colorectal surgeons in France for SFCs. In their study, a total of 190 out of 420 surgeons participated with a response rate of 45%. In the present study, SC was reported by 36.2% surgeons followed by STC (32.8%) and LHC (31%). Manceau et al. [10] in their study reported that the preferred procedure for SFCs among surgeons was SC (70%), followed by LHC (17%) and STC (13%).

In the present study, almost all the three procedures were performed equally, whereas SC was the most preferred surgical procedure reported by Manceau et al. [10]. Since all the variables involved here are categorical, a chi-square test was used to compare the results of the two studies. The test results showed that there was a statistically significant difference between the two studies with a p-value of 0.001 (X2 = 22.223). The comparison of surgical procedures between these two studies is given in Table 5.

**Table 5:** Comparison of surgical procedures between the present study and Manceau et al. Citation: Manceau et al. [10]

Name of surgical procedure	Results of the present study	Results of Manceau et al. study	X^2^	P-value
Splenic flexure colectomy	21 (36.2%)	133 (70%)	22.223	0.001^*^
Subtotal colectomy	19 (32.8%)	25 (13%)		
Left hemicolectomy	18 (31.0%)	32 (17%)		
Total	58 (100%)	190 (100%)		

Comparison of surgical approaches between the two studies

In the present study, laparoscopy was the most preferred surgical approach among the surgeons for doing procedures on SFCs reported by 49 (84.5%), followed by open surgery reported by five (8.6%) and hand-assisted laparoscopy reported by four (6.9%). Manceau et al. in their study had reported laparoscopy (63%), followed by laparotomy (31%) and hand-assisted laparoscopy (6%), as the most preferred surgical approaches preferred by surgeons. Since all the variables involved here were categorical and one of the cells in the table has an observed/expected count of less than 5, Fisher’s exact test was used to compare the results between two studies. The differences in the surgical approaches were found to be statistically significant with a p-value of 0.001. The comparison of surgical approach between the two studies is given in Table 6.

**Table 6:** Comparison of surgical approaches between the present study and Manceau et al. Citation: Manceau et al. [10]

Name of surgical procedure	Results of the present study	Results of Manceau et al.'s study	P-value
Laparoscopy	49 (84.5%)	120 (63%)	0.001*
Open surgery	5 (8.6%)	59 (31%)	
Hand assisted laparoscopy	4 (6.9%)	6 (11%)	
Total	58 (100%)	190 (100%)	

Comparison of SFC prognosis as felt by the surgeons in the two studies

In the present study, 31 (53.4%) surgeons felt that SFCs have a worse prognosis than other colonic tumors, whereas in the study done by Manceau et al., 29% of the responders thought that tumors of the splenic flexure had a worse prognosis in comparison with other colonic sites. The chi-square test was used here to compare the results between two studies as the variables were categorical. The test results showed that a higher percentage of surgeons in the present study felt that SFCs have a worse prognosis than Manceau et al.'s study, and the difference was found to be statistically significant with a p-value of 0.001 (X2 = 11.776). The comparison of tumor prognosis as felt by the surgeons in the two studies is given in Table 7.

**Table 7:** Comparison of splenic flexure colon cancer (SFC) prognosis as felt by surgeons in the present study and Manceau et al. Citation: Manceau et al. [10]

Perceived tumor prognosis	Results of present study	Results of Manceau G et al study	X^2^	P value
Worse	31 (53.4%)	55 (29%)	11.776	0.001*
Neutral	27 (46.6%)	135 (71%)		
	58 (100%)	190 (100%)		

## Discussion

The standard treatment for colon cancer is surgical resection, which depends greatly on lymphovascular drainage of the tumor and its location [6]. There remains much debate and varying opinions among specialists regarding the treatment of SFCs because of their variable lymphovascular drainage [11]. In this study, we compared our results with those of Manceau et al. [10] who conducted a study on the surgical practices of colorectal surgeons in France for SFCs. In their study, 190 of 420 surgeons participated, with a response rate of 45%. We wanted to compare our current study regarding surgical procedures, surgical approaches, and SFCs prognosis, as perceived by surgeons, with those of the study conducted in France by Manceau et al. [10] Among the surgeons in the present study, 36.2% preferred splenic flexure colectomy, followed by subtotal colectomy (32.8%), and left hemicolectomy (31%). According to Manceau et al. [10], 70% of surgeons prefer SFCs, followed by left hemicolectomy (17%) and subtotal colectomy (13%). In the present study, almost all three procedures were performed equally. Because all the variables involved here were categorical, the chi-square test was used to compare the results of the two studies. The test results showed a statistically significant difference between the two studies, with a p-value of 0.001 (χ2 = 22.223). A comparison of the surgical procedures between the two studies is presented in Table 5. This variation can be explained by the different training schools and backgrounds of the colorectal surgeons in Saudi Arabia.

In the present study, laparoscopy was the most preferred surgical approach among surgeons for performing procedures on SFCs (84.5%, n = 49), followed by open surgery (8.6%, n = 5) and hand-assisted laparoscopy (6.9%, n = 4). However, in the study by Manceau et al. [10], 63% of the surgeons reported favoring laparoscopy, followed by laparotomy (31%) and hand-assisted laparoscopy (6%) as the preferred surgical approach. Because all the variables involved were categorical and one of the cells in the table had an observed/expected count of less than five, Fisher’s exact test was used to compare the results between the two studies. The differences in surgical approaches were statistically significant (p = 0.001).

In the present study, 53.4% (n = 31) of the surgeons thought that patients with splenic flexure tumors have a worse prognosis than those diagnosed with other colonic tumors, which is in contrast to the 29% of respondents in the study by Manceau et al. [10] The chi-square test showed that the difference was statistically significant, with a p-value of 0.001 (χ2 = 11.776).

In another study conducted among members of the Association of Coloproctology of Great Britain and Ireland, there was a clear preference for extended right hemicolectomy (63%), with segmental resection coming in last (15.14%) [9]. Rega et al. compared three surgical procedures for patients with SFCs and found no differences in the overall survival rates of patients. Segmental splenic flexure resection was favored over alternative procedures because of the evidence provided for its oncological sufficiency [3]. Furthermore, there is no proof that extended resections are more effective than segmental resections, and they are associated with risks such as organ loss and frequent bowel movements [12]. Disagreements among colorectal surgeons in Saudi Arabia are likely attributable to variations in training and experience.

Regarding the surgical approach, similar to Huang et al. [13], laparoscopy was the most preferred procedure among the surgeons in our study (84.5%), compared to 63% and 50% of surgeons in other countries [9,10]. In contrast to open surgery, the laparoscopic removal of SFCs yields comparable oncological results with improved short- and long-term outcomes [14]. The rate of conversion to open surgery was similar to that of other colorectal surgeries [14]. There was a strong consensus among respondents in favor of using stapler anastomosis rather than hand sewing, with moderate agreement in preferring stapled side-to-side anastomosis configurations. By contrast, hand sewing is preferred in the United Kingdom and France at 62% and 51%, respectively [9,10].

Liu et al. found that staplers improved the surgical duration, recovery time, and complication rate in a study of 499 patients with gastrointestinal tumors [15]. Compared with the prognosis of patients with other colorectal carcinomas, those with SFCs have a poor outlook because of the risk of obstruction and diagnosis at an advanced stage [16,17]. However, other studies have stated that there is no difference in the survival rate of patients with SFC compared to those with other colonic segments [5,18].

 In concordance with other studies [9,10], 53% of the respondents in our study believed that patients with SFCs are more likely to have a poor prognosis compared to patients with other segmental cancers because they are diagnosed at an advanced stage and inadequate lymph node dissection is often performed.

An analysis from the American College of Surgeons-National Surgical Quality Improvement Program reviewed more than 3,000 patients with SFCs between 2012 and 2018 [19]. Elective splenic flexure colectomy in nonmetastatic cases appears adequate and feasible via laparoscopy, reduces operating time, has comparable postoperative morbidity (anastomotic leakage), yields good histological results, and has similar oncological outcomes.

Study limitation

This cross-sectional survey only comprises members of SSCRS. Although the sample size was small, it provided insights into their present approach and perspective on handling the challenging SFCs.

## Conclusions

In contrast to France, where segmental colectomy is the favored choice for surgical treatment of SFCs among colorectal surgeons, in Saudi Arabia, segmental colectomy, subtotal colectomy, and left hemicolectomy are performed with a similar frequency for managing SFCs. Colorectal surgeons in Saudi Arabia and France have significantly different attitudes toward managing SFCs.

Colorectal surgeons have not yet reached a consensus on the optimal approach for managing patients with SFCs. Therefore, it is crucial for the surgical community in Saudi Arabia to reach an agreement by organizing a series of expert meetings and efforts to build consensus.

## Appendices

Questions on the professional activities of colorectal surgeons

1- What type of colectomy do you usually perform electively in a patient with colon cancer located between the left third of the transverse colon and the proximal part of the descending colon? (Single answer)

o   Splenic flexure colectomy (or high left colectomy)

o   Subtotal colectomy (or extended right

o   Left hemicolectomy.

o   Total colectomy

o   Other (please specify)

2- Which blood vessel(s) do you ligate at its(their) origin(s) during the procedure?

o   Ileocolic vessels

o   Right colic vessels

o   Middle colic vessels

o   Left colic vessels.

o   Sigmoid vessels

o   Inferior mesenteric artery

o   Inferior mesenteric vein at the inferior border of the pancreas

o   Left branch of middle colic artery

3- Do you routinely use any specific method(s) to help with lymphadenectomy?

o   None

o   Sentinel lymph node mapping

o   CT with vascular reconstruction

o   Other (please specify)

4- Between which digestive segments do you perform the anastomosis at the end of the procedure? (Single answer)

o   Transverse colon and descending colon

o   Transverse colon and upper rectum

o   Terminal ileum and descending colon

o   Terminal ileum and sigmoid colon

o   Terminal ileum and upper rectum

o   I do not perform an anastomosis during the procedure

5- What type of anastomosis do you perform? (Single answer)

o   Hand-sewn end-to-end anastomosis

o   Stapled end-to-end anastomosis.

o   Hand-sewn side-to-end anastomosis

o   Stapled side-to-end anastomosis

o   Hand-sewn side-to-side anastomosis

o   Stapled side-to-side anastomosis.

o   Hand-sewn end-to-side anastomosis

6- Which surgical approach do you prefer for this type of procedure? (Single answer)

o   Laparoscopy

o   Open surgery

o   Hand-assisted laparoscopy

o   robotic

7- Regarding the greater omentum, what do you do? (Single answer)

o   Complete preservation of the greater omentum

o   Left partial omentectomy.

o   Total omentectomy preserving the vascular perigastric arcade.

o   Total omentectomy including the vascular perigastric arcade

8- What other procedure do you usually perform during the surgery?

o   None

o   Intraoperative colonic irrigation

o   Diverting stoma

o   Splenectomy

o   Left pancreatectomy with splenectomy.

o   Other (please specify)

9- Do you think these tumors have a worse prognosis than other colonic tumors?

o   Yes

o   No

10- If yes, for which reason(s)?

o   Insufficient lymph node dissection

o   Diagnosis at a more advanced stage

o   Other (please specify)

11- Are you practicing laparoscopic colorectal procedures for these tumors?

o   Yes

o   No

12- For obstructing left-sided colonic carcinomas, your preference would be:

o   Segmental left colectomy

o   Extended right colectomy

o   Resection with stoma o colostomy

o   Other

13- If you were to perform a resection of the obstruction of left colon cancer, would you perform it?

o   On-table lavage

o   Covering stoma

o   anastomosis

14- Have you had an internal herniation following laparoscopic left hemicolectomy?

o   Yes

o   No
